# Double Whammy: Rare Case of Infected Chronic Seroma Due to Bacterial Translocation From Biliary Sepsis

**DOI:** 10.7759/cureus.19044

**Published:** 2021-10-25

**Authors:** Katrina Ng, Adrian Teo

**Affiliations:** 1 General Surgery, Sir Charles Gairdner Hospital, Perth, AUS; 2 General Surgery, Armadale Health Service, Perth, AUS

**Keywords:** acute cholangitis, sepsis, hernia repair, bacterial translocation, seroma

## Abstract

We present a case of infected chronic seroma post ventral hernia repair using the Rives-Stoppa technique likely from bacterial translocation from ascending cholangitis. After definitive treatment with endoscopic retrograde cholangiopancreatography (ERCP) and drainage of obstructed gallstones, she continued to show signs of sepsis. Percutaneous drainage of seroma was diagnostic for infection, where *Escherichia coli* (*E. coli*) was cultured and coupled with IV antibiotics, her infection was treated. To the best of our knowledge, this is the first case of seroma infection from biliary sepsis, and there are no cases of infected seroma from a secondary infection in the literature.

## Introduction

The late onset of deep surgical site infection after hernia repair is rare. Incidence of late infection post abdominal wall hernia repair in a case series of 2666 was 0.3% (8/2666) [1]. There are no reported cases of infected seroma from bacteria translocation related to septicemia from a distant origin, and particularly there is no literature on infected seroma after sepsis.

This paper aims to report a case of infected chronic seroma secondary to ascending cholangitis.

## Case presentation

**Figure 1:**
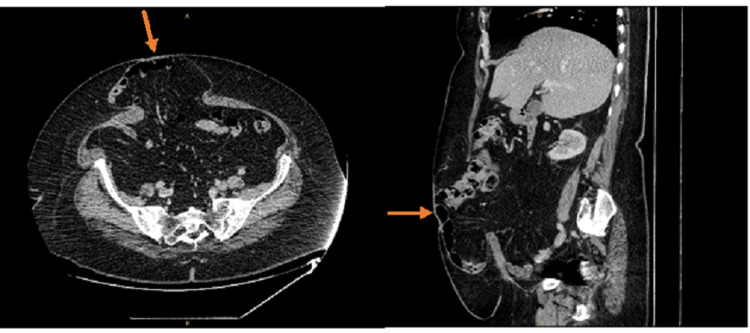
Pre-op CT abdomen prior to incisional hernia repair. Axial view on left, midline sagittal view on the right. Orange arrows indicate hernia.

A female in her 70s presented with a four-day history of fevers, right upper quadrant and epigastric abdominal pain associated with vomiting, loose stools, and dark urine. Significant past medical history includes previous open right hemicolectomy secondary to low-grade mucinous appendiceal neoplasm, asthma, type 2 diabetes mellitus, chronic kidney disease, gastroesophageal reflux, hypertension, previous hysterectomy, osteoarthritis, and morbid obesity with a BMI of 43. She developed a large midline incisional hernia with intestinal content a year after right hemicolectomy. She had undergone an open incisional hernia repair with mesh placement in the retro-rectus plane (Rives-Stoppa) in November 2019, a year prior to the current presentation. A CT abdomen done as part of colon cancer surveillance four months after repair incidentally showed a large seroma (Figure 1) but she was asymptomatic and had no signs of infection. After discussion with the patient and her treating surgeon, it was decided not to be treated as there was a potential risk of introducing infection which might lead to mesh explantation in the worst-case scenario.

On examination, she was febrile to 38.2˚C and other vital observations were normal. Clinically, she did not appear jaundiced. She was tender in the epigastric and right upper quadrant with a positive Murphy’s sign. There were no signs of cellulitis on the abdominal wall.

Laboratory investigations revealed raised inflammatory markers with leucocytosis (13.83*109/L) and C-reactive protein (CRP) of 430 mg/L. She had deranged liver function tests with a total bilirubin of 45 µmol/L, alanine aminotransferase (ALT) 133 U/L, gamma-glutamyl transferase (GGT) 548 U/L, and alkaline phosphatase (ALP) of 209 U/L. Blood culture and urine culture had no growth and chest X-ray did not show pneumonia.

**Figure 2:**
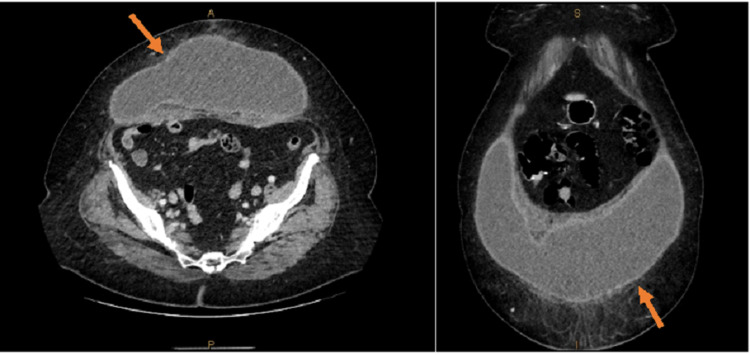
CT abdomen (axial slice on left, sagittal slice on right) taken a year after hernia repair with chronic seroma (orange arrows).

The CT abdomen revealed an obstructed biliary tree with a distal common bile duct (CBD) calculi and dilated CBD of 10 mm. The gallbladder contained multiple small calculi. A large horseshoe-shaped seroma in the anterior abdominal wall anterior to the rectus abdominis of similar size was again noted (Figure 2). It measured 23 cm axially, 9 cm in depth, and 22 cm craniocaudally.

She was started on broad-spectrum antibiotics, IV ceftriaxone (1 gm twice a day [BD]) and metronidazole (500 mg three times a day [TDS]). She underwent endoscopic retrograde cholangiopancreatography (ERCP) and sphincterotomy on day 3. Several stones were extracted with a balloon and the final occlusion cholangiogram was clear. Biochemical markers prior to ERCP showed white cell count (WCC) of 11.57*109/L, CRP 441 mg/L, total bilirubin of 64 µmol/L, ALT of 107U/L, ALP of 477 U/L, and GGT of 662 U/L. She felt clinically well the day after ERCP and remained afebrile. Laboratory investigations day after ERCP showed WCC of 17.42*109/L, CRP of 259 mg/L, total bilirubin of 22 µmol/L, ALT of 56 U/L, ALP 296 U/L, and GGT 358 U/L.

**Figure 3:**
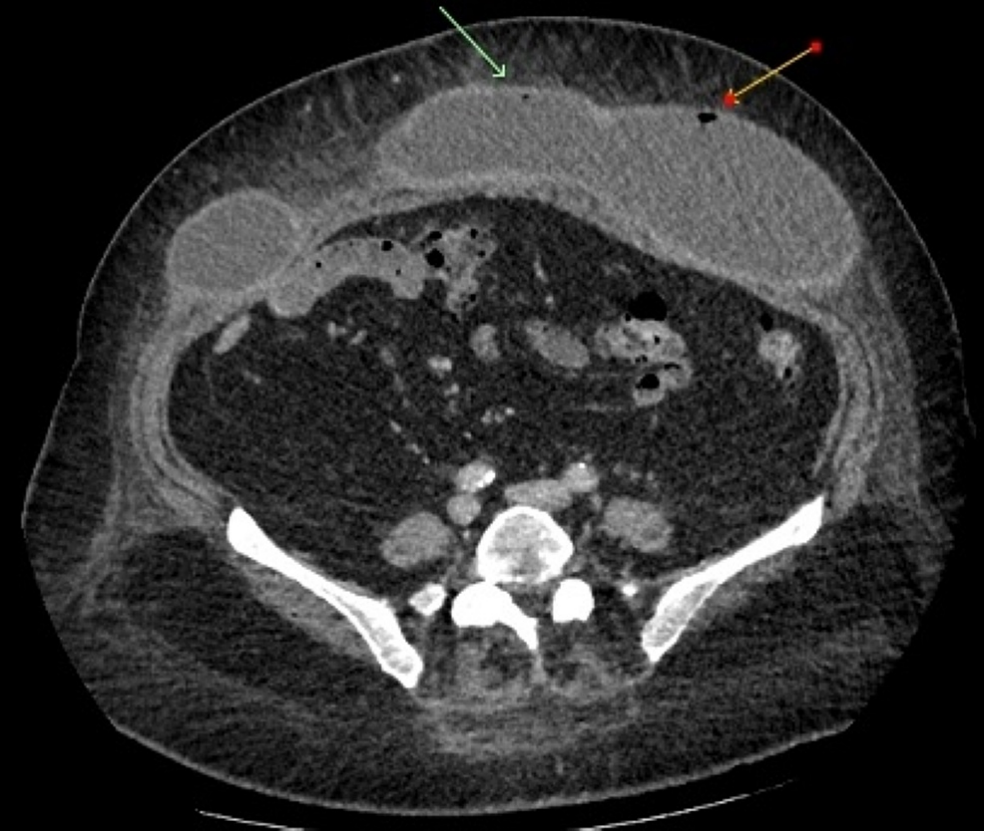
CT abdomen post ERCP (axial slice). Arrows pointing to gas locules within seroma.

She developed fevers day 2 post ERCP. On examination, she remained tender in the right upper quadrant with no signs of cellulitis on the abdominal wall. She was nontender in the central and lower abdomen. CT and ultrasound of the abdomen were repeated to exclude a complication from ERCP such as perforation or cholecystitis. It was found to have interval development of gas locules in the seroma (Figure 3). On ultrasound scan, the seroma had thickened walls and demonstrated internal septations. There was some edema noted in the overlying subcutaneous fat. Repeat blood and urine cultures were negative, and chest X-ray did not demonstrate infection.

**Figure 4:**
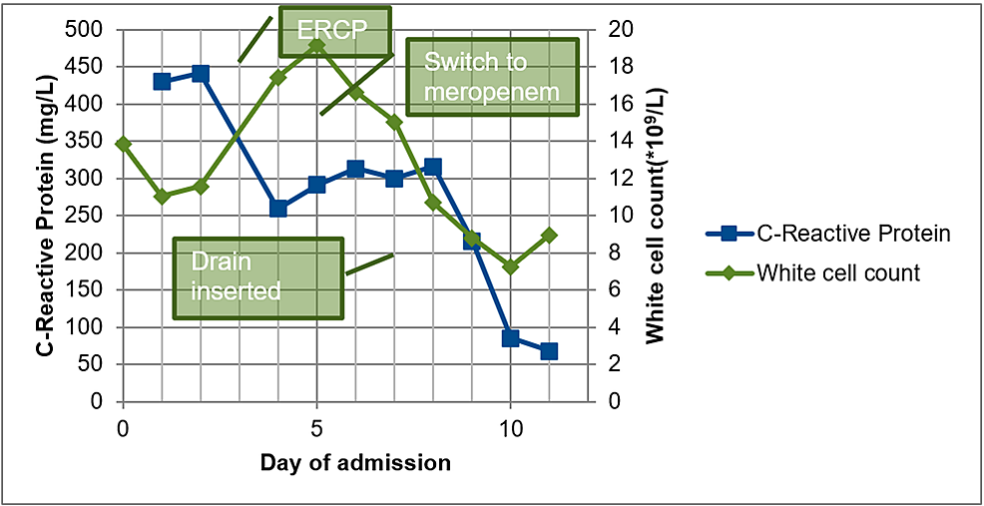
Inflammatory markers trend (bubbles indicating the day of intervention). ERCP: Endoscopic retrograde cholangiopancreatography.

**Figure 5:**
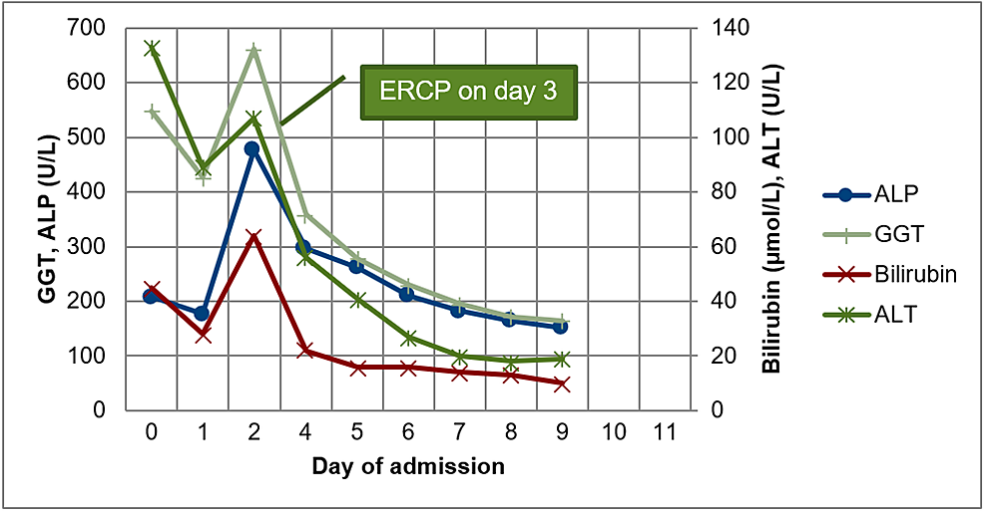
Liver function tests. ALT: Alanine aminotransferase; ALP: Alkaline phosphatase; ERCP: Endoscopic retrograde cholangiopancreatography; GGT: Gamma-glutamyl transferase.

**Figure 6:**
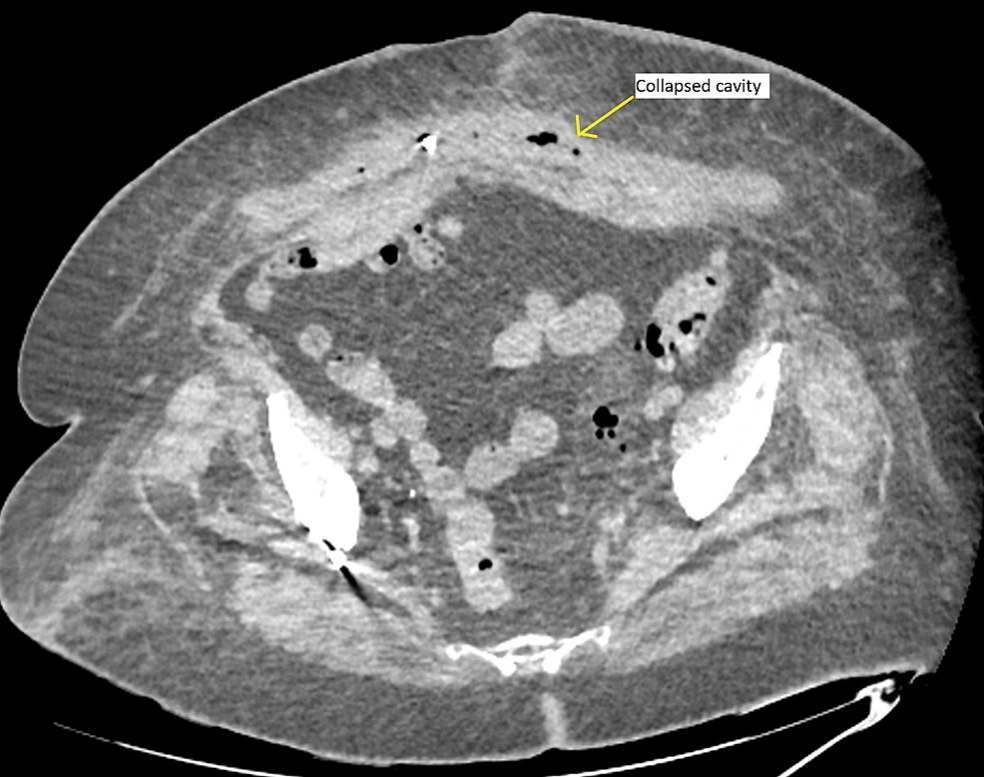
CT abdomen post drainage (axial slice).

Her antibiotics were changed to IV meropenem (1g TDS), but after two days she continued to spike fevers, and her inflammatory markers WCC and CRP continue to rise (Figure 4), while liver function tests continued to improve (Figure 5). She underwent ultrasound-guided drainage of the collection and placement of a pigtail catheter which contained blood-stained purulent fluid. A total of 2 L of fluid was drained. The culture of the fluid grew Escherichia Coli (E. coli) sensitive to multiple types of antibiotics including ceftriaxone and meropenem, and antibiotics were changed back to ceftriaxone (1g BD). A repeat CT was obtained after five days which showed near complete drainage of the collection (Figure 6) and the catheter was removed. She had no further fevers and serum leucocytes were normal at 9.86 mmol/L and CRP was down trending at 102 from a peak of 313. She was discharged home with a further five days of oral ciprofloxacin.

## Discussion

Seroma formation after ventral hernia repair is common, with incidence approximately 30% [2], and most are sterile and resolve spontaneously. The reason for this is multifactorial, but the main cause is tissue dissection of the anterior abdominal wall leading to reactive inflammation and accumulation of lymphatic fluid and blood [3]. This is further exacerbated if the hernia was large and significant potential space is created after the repair. Systemic infection leading to contamination of a previously sterile collection is demonstrated in this case. While in this case, blood cultures were not positive, the fact that *E. coli*, a common enteric organism was grown in the seroma fluid makes it likely that the source of infection was from biliary sepsis.

Infection is the most severe of complications from seroma formation as in the worst-case scenario it could lead to re-operation and explantation of mesh if the infection involves the mesh. Incidence of mesh infection is up to 10% in ventral hernia repair and risk factors are advanced age, American Society of Anesthesiologists (ASA) score greater than 2, tobacco smoking, obesity, and inadequate glycemic control in patients with diabetes mellitus. [4] The mainstay for the treatment of superficial infection is systemic antibiotics and drainage of collection, with consideration of surgical debridement and mesh removal if the patient fails conservative management [1].

It has been reported that deep-seated infections can occur months after surgery and sometimes even up to 4.5 years [5]. Delikoukos S et al. reported five cases in 1452 patients with delayed infection and they all primarily sought medical attention for symptoms related to the hernia site [5]. This could arise as a complication of a persisting collection [6]. The success of percutaneous drainage of collection from mesh infection is mixed. Kuo YC et al. reports salvaging mesh repairs in 9/14 patients [7], while Chen T et al. report only 1/8 patients who recovered with antibiotics and drainage [1].

There have been reports of prostheses such as breast implants and joint prostheses being infected from a secondary source [8,9]. However, there have not been any reports of mesh or seroma infection seeding from secondary infection sites. Bacteria implicated in previous reports included both Gram-positive bacteria such as *Staphylococcus aureus* and *Staphylococcus epidermidis* and Gram-negative organisms such as *Bacteroides fragilis* and *Pasteurella multocida*. Bacteraemia could be introduced through interruption of the natural skin barrier or from the gut with peritonitis.

The success of treatment with percutaneous drainage without need for mesh explantation was increased with an underlay repair as compared to an onlay repair in a study done by Bueno-Lledó J et al. [10]. While this could be secondary to a reduction in exposure to wound flora and protection from the thickness of muscle and adipose tissue, this would not apply in late infection after the superficial wound has healed. As seromas form in a potential space of least resistance, this would be in the subcutaneous layer where the previously herniated contents were. Due to the abdominal wall defect, the ventral fascia closure is tight and therefore leaves less space for a seroma to form. The closure of the ventral fascia also creates a barrier between the mesh and the seroma, which could prevent the need for the explantation of the mesh [11].

## Conclusions

This case suggests that a potential risk factor of late-onset infected seroma is systemic sepsis, and a pre-existing seroma/collection should be considered as a source of sepsis even if there are no localized symptoms or signs. Percutaneous drainage and antibiotic treatment were effective in this patient. Placement of mesh in the retro-rectus space with closure on anterior fascia potentially prevented the need for mesh explantation.
